# Potential for human consumption of fermented millet (*Kunun zaki*) to reduce the prevalence of selected antimicrobial resistance genes in human fecal samples

**DOI:** 10.7717/peerj.21495

**Published:** 2026-07-14

**Authors:** Haruna Jimoh Audu, Marion Byonanebye, Nyah Allen, Olumuyiwa Samuel Alabi, Funmilola A. Ayeni

**Affiliations:** 1 Pharmaceutical Microbiology and Biotechnology, Faculty of Pharmacy, University of Ibadan, Ibadan, Oyo State, Nigeria; 2 Environmental and Occupational Health, Indiana University, Bloomington, IN, United States of America; 3 Biology, Indiana University, Bloomington, IN, United States of America

**Keywords:** Dietary intervention, Antibiotic resistance genes, Gut microbiota, Fermented beverages, Resistome, Dietary intervention

## Abstract

**Background:**

Antimicrobial resistance (AMR) poses a major global health challenge, with the human gut microbiota acting as a key reservoir for resistance genes. Traditional fermented foods may influence microbial gut dynamics and AMR gene carriage.

**Objective:**

This study evaluated the occurrence of AMR genes in participants from Nigeria and, possible effect of consumption of two Nigerian fermented beverages, *Kunun aya* and *Kunun zaki* on the prevalence and distribution of selected AMR genes in the human gut microbiota.

**Methods:**

In this exploratory pilot study, 36 healthy volunteers from Abuja, North Central Nigeria were assigned to three groups: *Kunun aya, Kunun zaki*, and control (no intervention). Participants consumed their assigned beverage daily for two weeks, followed by a two-week washout phase. Fecal samples collected at the three timepoints were tested for 11 AMR genes using conventional Polymerase Chain Reaction (PCR). Treatment effects were expressed as percentage point changes with 95% confidence intervals. Fisher’s exact test was used to assess baseline prevalence differences across groups, and Pearson correlation coefficients were used to assess gene co-occurrence patterns.

**Results:**

At baseline (*n* = 36), dfrA was the most prevalent gene (77.8%), followed by blaTEM (41.7%), mefA/E (38.9%), ermB (33.3%), qnrA (30.6%), and blaCTX-M (13.9%). Beverage consumption was associated with divergent patterns: *Kunun zaki* was associated with reductions in four of six genes (mean change –11.7 percentage points), with the largest decreases observed for qnrA (–66.7 percentage points) and dfrA (–23.3 percentage points), In contrast, *Kunun aya* was associated with increases in five of six genes (mean change +18.1 percentage points), including blaTEM (+38.9 percentage points) and ermB (+30.6 percentage points) though no increase was significant. The control group showed minimal changes (mean –0.8 percentage points). No statistically significant sex-based differences in gene prevalence were observed.

**Conclusions:**

There is high occurrence of dfrA gene in the studies population, concurrent with the uncontrolled use of trimethoprim in the study environment. However, consumption of *Kunun zaki* was associated with reduced prevalence of specific AMR genes, particularly dfrA and qnrA, *while Kunun aya* was associated with increases underscoring the duplibiotic potential of fermented drinks on the gut ARG abundance. These preliminary findings support the exploration of culturally accepted fermented foods as complementary strategies to combat AMR in low and middle income countries. However, given the pilot design, small sample size and exploratory design of this study, larger studies are needed to confirm these preliminary results.

## Introduction

Antimicrobial resistance (AMR) is one of the most critical global health threats of the 21st century, currently causing approximately 700,000 deaths annually (Milkowska-Shibata, 2020; (Jasovsky et al., 2016). This number is projected to surge to 10 million per year by 2050 without effective interventions (Milkowska-Shibata, 2020; (Jasovsky et al., 2016). In the US alone, over 210 million outpatient oral antibiotic prescriptions are issued annually, fueling global alterations in the gut microbiota (Le Bastard et al., 2018; Dahiya & Nigam, 2023; Guarner et al., 2024). Rising rates of treatment failure and mortality due to drug-resistant infections highlight the urgent need for sustainable, innovative strategies to curtail the spread of antibiotic resistance genes (ARGs) (Elshobary et al., 2025). This issue is severe in low- and middle-income countries (LMICs), where overuse and misuse of antibiotics, inadequate sanitation, limited diagnostic infrastructure, and weak surveillance systems accelerate the spread of AMR (Iskandar et al., 2020; Murugaiyan et al., 2022; Singha, Singh & Soni, 2024).

The human gut microbiota has emerged as a key reservoir and transmission hub for ARGs. Antibiotics, especially those administered orally or excreted *via* the biliary route, disrupt gut microbial communities and promote the expansion of the resistome (Pilmis, Le Monnier & Zahar, 2020; Call et al., 2020). Moreover, emerging evidence shows that even non-antibiotic drugs can alter gut microbiota structure and function, further contributing to resistance gene proliferation (Li et al., 2025; Bastard et al., 2021; Lathakumari et al., 2024).

ARGs in the gut microbiota are capable of long-term persistence, even in the absence of antibiotic pressure, due to vertical and horizontal gene transfer mechanisms (Deshpande et al., 2025; Crits-Christoph et al., 2022; Baek et al., 2025; Lopatkin et al., 2017). This calls for interventions that go beyond conventional antibiotic stewardship. Fecal microbiota transplantation (FMT) has shown promise in treating recurrent *Clostridium difficile* infections and restoring microbial diversity, but concerns remain over the possible transmission of multidrug-resistant organisms (Park & Seo, 2021; Gupta, Mullish & Allegretti, 2021; Liu & Wang, 2020). Even autologous FMT has yielded mixed results in reducing ARG burdens (Defilipp et al., 2019). These limitations call for safe, culturally relevant alternatives. Diet has become increasingly recognized as a key modulator of gut microbiota. Diets rich in fiber, prebiotics, and particularly fermented foods have been shown to improve microbial diversity and immune function, while also potentially reducing ARG carriage (Rinninella et al., 2023; Perrone & Angelo, 2025; Wastyk et al., 2021). Traditional fermented foods contain live microbes and bioactive compounds that compete with resistant organisms and restore microbial balance (Nagarajan, Rajasekaran & Venkatachalam, 2022). Despite growing interest in dietary strategies to mitigate AMR, few studies have evaluated their real-world effects, particularly in LMICs.

Fermented foods have been consumed for centuries and are increasingly acknowledged for their ability to modulate the gut microbiota, inhibit pathogenic colonization, and support mucosal immunity (Leeuwendaal et al., 2022; Crowder et al., 2023; Balasubramanian et al., 2024). While most research has focused on Western fermented foods such as yogurt, kefir, and kimchi, the impact of African fermented beverages remains underexplored. These traditional foods, widely consumed and embedded in local cultures, may represent low-cost and effective strategies for AMR mitigation especially in LMiCs where the prevalence of AMR is high (Anukam & Reid, 2009; Obafemi et al., 2022).

In Nigeria, *Kunun aya* (tigernut drink) and *Kunun zaki* (millet- or sorghum drink) are two widely consumed traditional beverages made *via* spontaneous fermentation involving lactic acid bacteria (LAB) and yeast (Ogunyemi et al., 2021; Olaoye, Ndife & Raymond, 2017). LAB from these beverages have shown antimicrobial activity and may influence ARG carriage through competitive exclusion and metabolite production (Albene et al., 2024; Choi et al., 2018; Adesemoye et al., 2025). However, evidence from *in vivo* human intervention studies assessing their impact on antimicrobial resistance gene (ARG) carriage in the gut microbiota remains limited.

In light of the potential of fermented foods as modifiable dietary interventions and their deep cultural acceptance, this study aimed to evaluate the occurrence of AMR genes in participants from Nigeria and, impact of daily consumption of *Kunun aya* and *Kunun zaki* on the prevalence and distribution of selected ARGs in the gut microbiota of healthy humans. We hypothesized that these beverages, due to their microbial content and fermentation-derived metabolites, could modulate high abindance of gut ARG carriage by suppressing resistant bacterial populations.

### Rationale for the study

Kunu contains beneficial bacteria as well as blended cereal (fibre) which makes it suitable to serve both as a probiotic through gut microbiota modulation, and prebiotic by stimulating the growth of other bacteria. The fermentation process in Kunun production involves lactic acid bacteria which may competitively exclude potential pathogenic bacteria and their associated resistance genes. Understanding how traditional fermented beverages affect the gut resistome is important for public health, particularly in regions where antimicrobial resistance is a growing concern and where these beverages are regularly consumed.

## Method

### Preparation of fermented drinks

*Kunun zaki* was prepared by soaking millet grains for 4–6 h to naturally ferment, then washed and milled into a slurry, which was divided into two equal portions. A separate spice paste was made by blending from four g of ginger, one g of cloves, and four g of dried potato. The blended spices were mixed with one portion of milled millet grains, then boiled water was added to the mixture to form a thick paste, which was cooled to room temperature. The second portion of milled millet was added to the cooled paste, followed by the addition of 50–100 ml of water at room temperature to achieve a servable consistency. The mixture was then sieved using a strainer cloth and served in bottles. *Kunun aya* was prepared by blending the following to a smooth consistency: 120 g of washed tiger nuts (soaked for 5–6 hous), four washed dates (seeds removed), 10 g of peeled coconut, four g of ginger and one g of cloves. The resulting slurry was sieved using a strainer cloth and serve in bottles.

### Study population and sample collections

**Figure 1:**
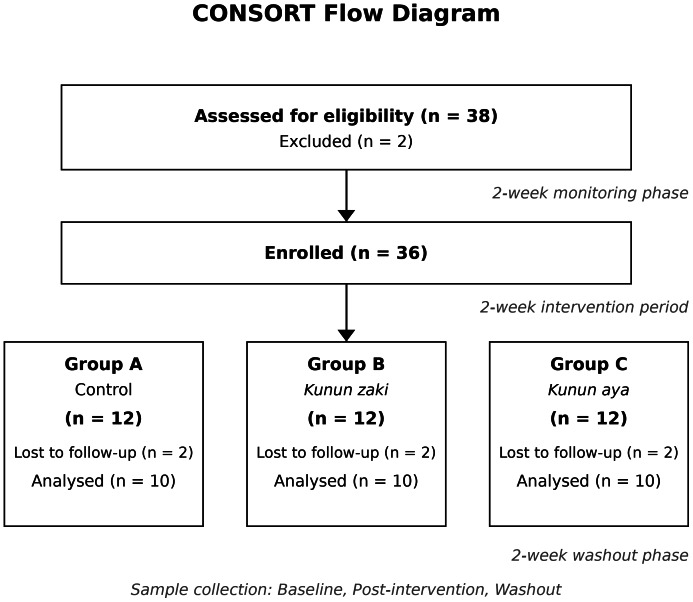
Consort flowchart. Enrolment, group allocation, and sample completion across the three study groups.

This quasi human intervention study was approved by the ethics committee of the Institute of Advanced Medical Research and Statistics, IAMRAT, UCH, Ibadan with assigned number UI/E *C*/21/0625. It was also registered as a clinical trial with the Pan African Clinical Registry with number PACTR202401853406618 on 12/01/2024 (https://pactr.samrc.ac.za). Recruitment of participants as well as intervention took place between January, 2024 and March 2024. All participants signed written informed consent to be part of this research. A total of 36 healthy human volunteers (20 females and 16 males), residing in Abuja North Central Nigeria and aged between 3 and 65 years, were recruited for this study. One minor aged 3 years was included with parental informed consent and assent gotten from the child. This inclusion was consistent with microbiome research practices, as the gut microbiome assumes an adult-like configuration by early childhood (Yatsunenko et al., 2012). Inclusion criteria required participants to be healthy individuals within this age range. Exclusion criteria included recent or ongoing use of antibiotics, intentional consumption of fermented products, the presence of gastrointestinal disorders such as diarrhea, inflammatory bowel disease, or irritable bowel syndrome, pregnancy or breastfeeding, and alcoholism or drug addiction within two weeks prior to the first sample collection. Eligible participants entered a two-week monitoring phase to ensure participants’ compliance with exclusion criteria, verified through a daily online 24-hour food and drug recall questionnaire while those who did not meet the criteria were excluded. After the monitoring phase, participants were assigned to either *Kunun aya*, *Kunun zaki* or control group (no intervention), (12 participants per group) corresponding to groups A, B and C, respectively. Due to the exploratory nature of this study, no formal power calculation was employed as data generated will be used to plan more adequately powered studies. Participants were assigned IDs by chance as they came, but no formal random sequence generation or allocation concealment method was used. Of the 36 participants enrolled (12 per group), six participants (two from each group) had incomplete longitudinal data due to inability to provide samples at all timepoints (Fig. 1). Analyses employed an available-case approach, including all participants with data at each timepoint, resulting in sample sizes of *n* = 36 at baseline, *n* = 35 at intervention, and *n* = 30 at washout.

The participants supplemented their habitual diet with 500 mL (50 cL) daily of one of the assigned beverages. This was followed by a two-week washout phase. Fecal samples were self-collected by the participants across the three phases: prior to beverage consumption (baseline), after two weeks of beverage consumption, and at two weeks post-intervention (washout phase). Upon collection, samples were immediately homogenized in a tube containing 30 ml of 97% pharmacy grade ethanol for 24-36 h to form a bolus. The bolus formed afterwards was then transferred to 50 ml tubes containing silica gel beads topped with a kim wipe. The silica beads acted as a desiccant by removing moisture from the fecal matrix. Samples were stored at room temperature in sealed containers with silica beads until DNA extraction. This silica bead-based desiccation method has been validated for preserving bacterial and fungal DNA integrity in samples and provides a cost-effective alternative to cold storage (Nsubuga et al., 2004; Smenderovac et al., 2025). DNA was extracted from silica-preserved fecal samples using the QIAamp DNA Stool Mini Kit (Qiagen, Hilden, Germany) according to the manufacturer’s instructions. Extracted DNA samples were stored at −20 °C until PCR analysis. DNA was extracted from fecal samples using the DNA Stool Mini Kit (Qiagen, Hilden, Germany) according to the manufacturer’s instructions. Extracted DNA samples were stored at −20 °C until further PCR analysis. PCR was done for AMR genes that are associated with antibiotics commonly used in Nigeria.

### PCR amplification of genes

All PCR reactions were performed in 20 µL volumes using a standard reaction mixture comprising 10 µL Master mix, 1 µL DNA template, 1 µL each of forward and reverse primers (or multiplex primer sets), and molecular-grade water to final volume. For multiplex reactions targeting multiple genes, primer volumes were adjusted accordingly while maintaining the 20 µL total reaction volume. Gene-specific thermal cycling conditions are detailed in Table 1. Reactions included a positive control (from our laboratory collection) and a negative control (molecular-grade water) to validate amplification specificity. Primer sequences, expected amplicon sizes, and references are provided in Table 2.

**Table 1:** PCR thermal cycling conditions for ARG. A table showing the PCR parameters set for amplifying the different genes.

**Gene (s)**	**Initial denaturation**	**Cycles**	**Denaturation**	**Annealing**	**Extension**	**Final extension**
blaTEM, blaSHV	94 °C, 5 min	30	94 °C, 30 s	50 °C, 30 s	72 °C, 90 s	72 °C, 10 min
blaCTX-M	94 °C, 5 min	30	94 °C, 30 s	56 °C, 1 min	72 °C, 60 s	72 °C, 10 min
dfrA	95 °C, 5 min	35	95 °C, 60 s	55 °C, 1 min	72 °C, 60 s	72 ° C, 10 min
qnrB, qnrS	94 °C, 5 min	32	94 °C, 45 s	53 °C, 45 s	72 °C, 60 s	72 °C, 10 min
qnrA, qepA	94 °C, 2 min	35	94 °C, 30 s	53 °C, 30 s	72 °C, 60 s	72 °C, 10 min
blaZ, ermA, ermB, mef (A/E)	95 °C, 5 min	30	95 °C, 1 min	59 °C, 1 min	72 °C, 90 s	72 °C, 10 min

**Table 2:** List of primers used. Table showing primers used for ARG identification, their sequences, amplicon sizes and annealing temperatures.

**Gene**	**Antibiotic resistance**	**Primer sequence (5′→3′)**	**Amplicon size**	**Annealing temp**
blaTEM	**Beta-lactams**: Penicillins, Cephalosporins (extended-spectrum)	F: GAGTATTCAACATTTTCGT R: ACCAATGCTTAATCAGTGA	857 bp	50 ^∘^C (30 s)
blaSHV	**Beta-lactams**: Penicillins, cephalosporins (extended-spectrum)	F: TCGCCTGTGTATTATCTCCC R: CGCAGATAAATCACCACAATG	768 bp	50 ^∘^C (30 s)
blaCTX-M	**Beta-lactams**: Cephalosporins, Penicillins	F: TTTGCGATGTGCAGTACCAGTAA R: CGATATCGTTGGTGGTGCCATA	543 bp	56 ^∘^C (1 min)
dfrA	**Trimethoprim**	F: CTTGTTAACCCTTTTGCCAGA R: TTGTGAAACTATCACTAATGGTAG	489 bp	55 ^∘^C (60 s)
qnrA	**Fluoroquinolones**:	F: ATTTCTCACGCCAGGATTTG R: GATCGGCAAAGGTTAGGTCA	516 bp	53 ^∘^C (30 s)
qnrB	**Fluoroquinolones**:	F: GATCGTGAAAGCCAGAAAGG R: ACGATGCCTGGTAGTTGTCC	469 bp	53 ^∘^C (45 s)
qnrS	**Fluoroquinolones**:	F: ACGACATTCGTCAACTGCAA R: TAAATTGGCACCCTGTAGGC	417 bp	53 ^∘^C (45 s)
qepA	**Fluoroquinolones**:	F: CTTCTCTGGATCCTGGACAT R: TGAAGATGTAGACGCCGAAC	720 bp	53 ^∘^C (30 s)
mef (A/E)	**Macrolides**:	F: CAATATGGGCAGGGCAAG R: AAGCTGTTCCAATGCTACGG	317 bp	59 ^∘^C (1 min)
ermA	**Macrolides, Lincosamides, Streptogramin B**	F: CCCGAAAAATACGCAAAATTTCAT R: CCCTGTTTACCCATTTATAAACG	590 bp	59 ^∘^C (1 min)
ermB	**Macrolides, Lincosamides, Streptogramin B**	F: TGGTATTCCAAATGCGTAATG R: CTGTGGTATGGCGGGTAAGT	745 bp	59 ^∘^C (1 min)
blaZ	**Beta-lactams**:	F: ACTTCAACACCTGCTGCTTTC R: TGACCACTTTTATCAGCAA	173 bp	59 ^∘^C (1 min)

### Visualization and quality control

All PCR products were analyzed by electrophoresis on 1.5% agarose gels stained with SYBR Safe dye and visualized under UV light, to confirm the presence and expected size of amplification products relative to a molecular weight DNA ladder.

### Statistical analysis

A >5% baseline ARG prevalence (before the participants took any intervention) was set for each gene analysis. Of the 11 genes initially screened, only six genes *i.e*, *dfrA, blaTEM, MefA/E, ermB, qnrA*, and *bla_CTX-M* exceeded the >5% baseline prevalence threshold and were retained for analysis. This cut off threshold helps to ensure that selected genes have sufficient representation across the study population to allow meaningful descriptive comparisons over time.

All statistical analyses were performed using R version 4.4.2 Descriptive statistics were used to summarize the prevalence and distribution of ARGs across interventions and timepoints. Treatment effects were expressed as percentage point differences between baseline and subsequent timepoints, with 95% confidence intervals estimated using t-distribution appropriate for small sample sizes (*df* = 8). Baseline prevalence of resistance genes was compared using Fisher’s exact test, and differences in gene prevalence by sex at baseline across intervention and control groups were also evaluated with Fisher’s exact test. For treatment effect analysis, effect sizes were calculated with 95% confidence interval. Gene co-occurrence patterns at baseline were assessed using Pearson correlation coefficients on individual-level presence/absence data for the six genes across all participants. Statistical significance was defined as *p* < 0.05.

## Results

### Baseline prevalence of AMR genes

**Figure 2:**
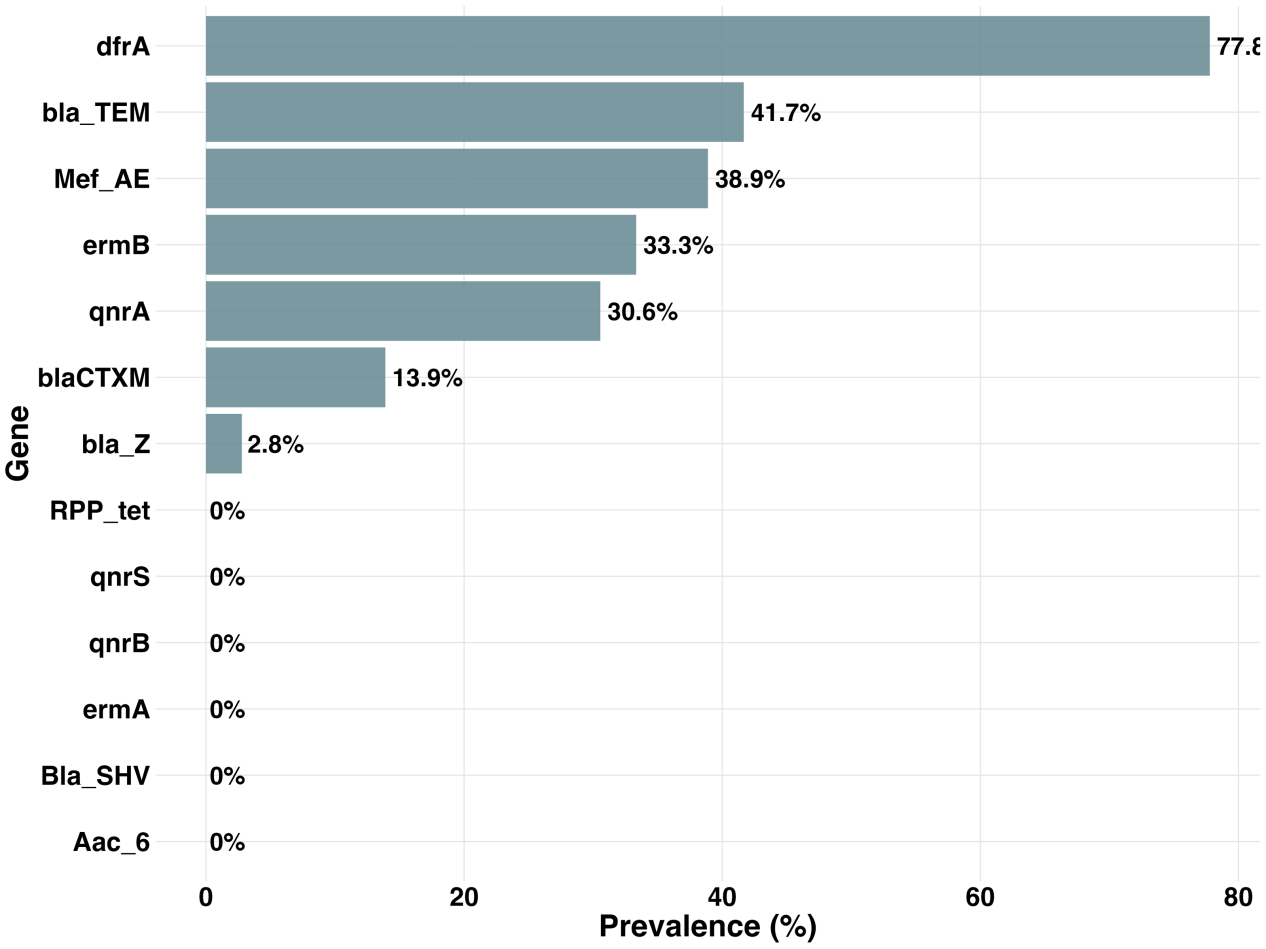
Baseline prevalence of antimicrobial resistance genes in gut microbiota. Prevalence of six ARGs detected at baseline (*n* = 36 participants) across all intervention groups. Genes are ordered by decreasing prevalence. Error bars represent 95% Wilson confidence intervals. ARG, antibiotic resistance gene.

At baseline, *dfrA* was the most prevalent ARG, detected in 77.8% of participants. This was followed by *blaTEM* (41.7%) and *mefA/E* (38.9%). The *ermB* gene was present in 33.3% of participants, while *qnrA* had a prevalence of 30.6%. *BlaCTX-M* was detected in 13.9% of individuals (Fig. 2). Baseline comparisons showed no significant differences in gene prevalence across intervention groups for most genes; however, baseline imbalances were observed for *dfrA* (*p* = 0.03) and *qnrA* (*p* = 0.008). To account for these baseline differences, we assessed intervention effects by examining within-group changes over time rather than direct between-group comparisons at individual timepoints.

### Longitudinal trends in gene prevalence

**Figure 3:**
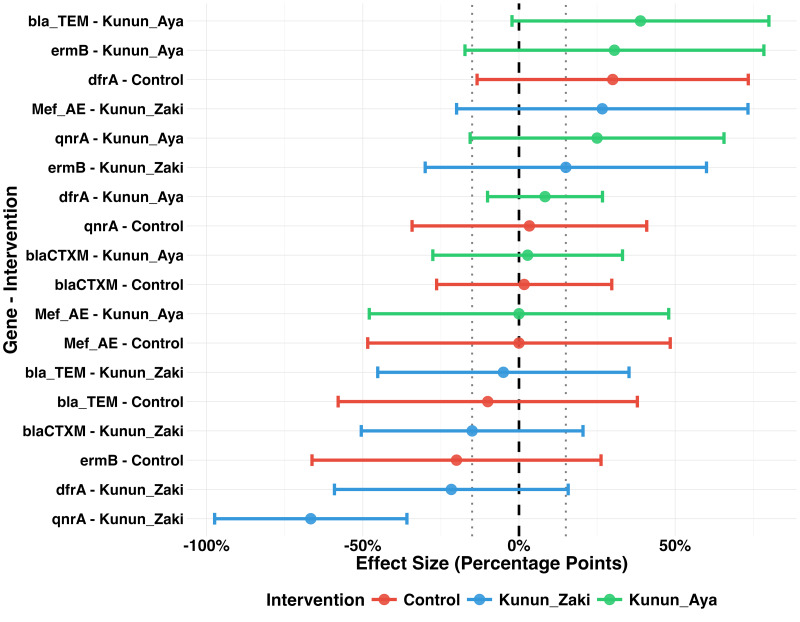
Forest plot of treatment effects. Forest plot showing percentage point changes in ARG prevalence from baseline to washout for each gene-intervention combination. Points represent effect sizes (prevalence at washout minus prevalence at baseline). Error bars represent 95% confidence intervals. Vertical dashed line at zero indicates no change. Kunun zaki (blue) was associated with predominantly negative effects (reductions), Kunun aya (green) with predominantly positive effects (increases), and control (red) with minimal changes.

**Figure 4:**
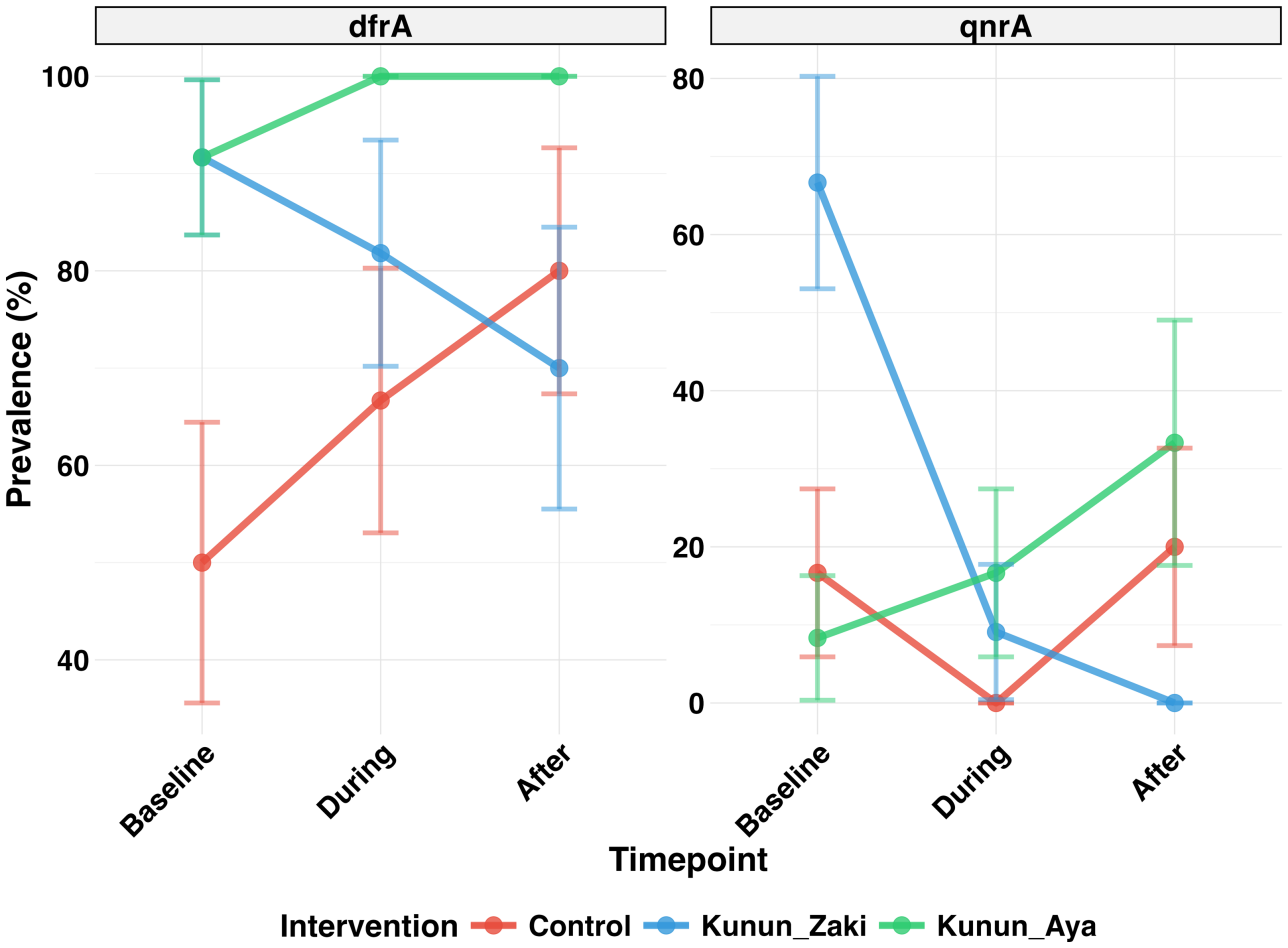
Gene prevalence for dfra and qnrA. Line plots showing prevalence trajectories for (A) dfrA and (B) qnrA across three study phases: baseline, intervention, and washout. Points represent prevalence at each timepoint with error bars indicating 95% Wilson confidence intervals. Lines connect the same intervention group across timepoints: Control (red), Kunun zaki (blue), and Kunun aya (green).

Sample sizes varied across timepoints due to incomplete sample collection: baseline *n* = 36, intervention phase *n* = 35, washout, *n* = 30. The associations between beverage consumption and ARG prevalence varied by tested ARG at baseline (timepoint 1), during intervention (Timepoint 2), and after washout (Timepoint 3) (Fig. 3). For the *Kunun zaki* group, the most notable chronological shift was observed for *qnrA*, which declined by 57.6 percentage points from baseline (66.7%) to post intervention (9.1%) and then reached 0% after washout (Fig. 4). *blaCTX-M* also decreased in prevalence from 25.0% at timepoint 1 to 18.2% at timepoint 2 (6.8 percentage point reduction), and 10.0% at timepoint 3 (15.0 percentage point reduction overall) in the *Kunun zaki* group. For *dfrA* gene, there was a decrease in prevalence in the *Kunun zaki* group from 91.7% at timepoint 1 to 81.8% at timepoint 2 (9.9 percentage point reduction), and 70.0% at timepoint 3 (21.7 percentage point reduction overall). *blaTEM* showed an initial increase from 25.0% to 27.3% at timepoint 2 (+2.3 percentage points), before declining to 20.0% at timepoint 3 (5.0 percentage point reduction overall). *ermB* increased from 25.0% at timepoint 1 to 36.4% at timepoint 2 (+11.4 percentage points), and 40.0% at timepoint 3 (+15.0 percentage points overall). *MefAE* decreased from 33.3% to 18.2% at timepoint 2 (-15.1 percentage points), before rising to 60.0% at timepoint 3 (+26.7 percentage points overall).

In contrast, the *Kunun aya* group showed a notable rise in *qnrA* prevalence from 8.3% at timepoint 1 to 16.7% at timepoint 2 (+8.4 percentage points), and 33.3% at timepoint 3 (+25.0 percentage points overall) (Fig. 3). *dfrA* prevalence also remained high in the *Kunun aya* group, rising from 91.7% at timepoint 1 to 100.0% at timepoint 2 and remaining at 100.0% at timepoint 3 (+8.3 percentage points overall). The largest increases were observed for *blaTEM*, which rose from 50.0% at timepoint 1 to 75.0% at timepoint 2 (+25.0 percentage points), and 88.9% at timepoint 3 (+38.9 percentage points overall). *ermB* increased from 25.0% at timepoint 1 to 33.3% at timepoint 2 (+8.3 percentage points), and 55.6% at timepoint 3 (+30.6 percentage points overall). *blaCTX-M* increased from 8.3% at timepoint 1 to 16.7% at timepoint 2 (+8.4 percentage points), and 11.1% at timepoint 3 (+2.8 percentage points overall). *MefAE* rose from 33.3% to 50.0% at timepoint 2 (+16.7 percentage points), before returning to 33.3% at timepoint 3 (no overall change).

The control group showed a near-neutral mean change (+0.8 percentage points), contrasting with the *Kunun zaki* intervention group (mean: −11.1 percentage points). *dfrA* increased from 50.0% at timepoint 1 to 66.7% at timepoint 2 (+16.7 percentage points) and 80.0% at timepoint 3 (+30.0 percentage points overall). *blaTEM* initially increased from 50.0% to 75.0% at timepoint 2 (+25.0 percentage points), before declining to 40.0% at timepoint 3 (−10.0 percentage points overall). *ermB* decreased from 50.0% at timepoint 1 to 25.0% at timepoint 2 (−25.0 percentage points), and 30.0% at timepoint 3 (−20.0 percentage points overall). *MefAE* showed a slight decrease from 50.0% to 41.7% at timepoint 2 (−8.3 percentage points), before returning to 50.0% at timepoint 3 (no overall change). *qnrA* declined from 16.7% at timepoint 1 to 0% at timepoint 2 (-16.7 percentage points), before rising to 20.0% at timepoint 3 (+3.3 percentage points overall). *blaCTX-M* remained relatively stable, increasing from 8.3% at timepoint 1 to 10.0% at timepoint 3 (+1.7 percentage points) (Fig. 3).

**Figure 5:**
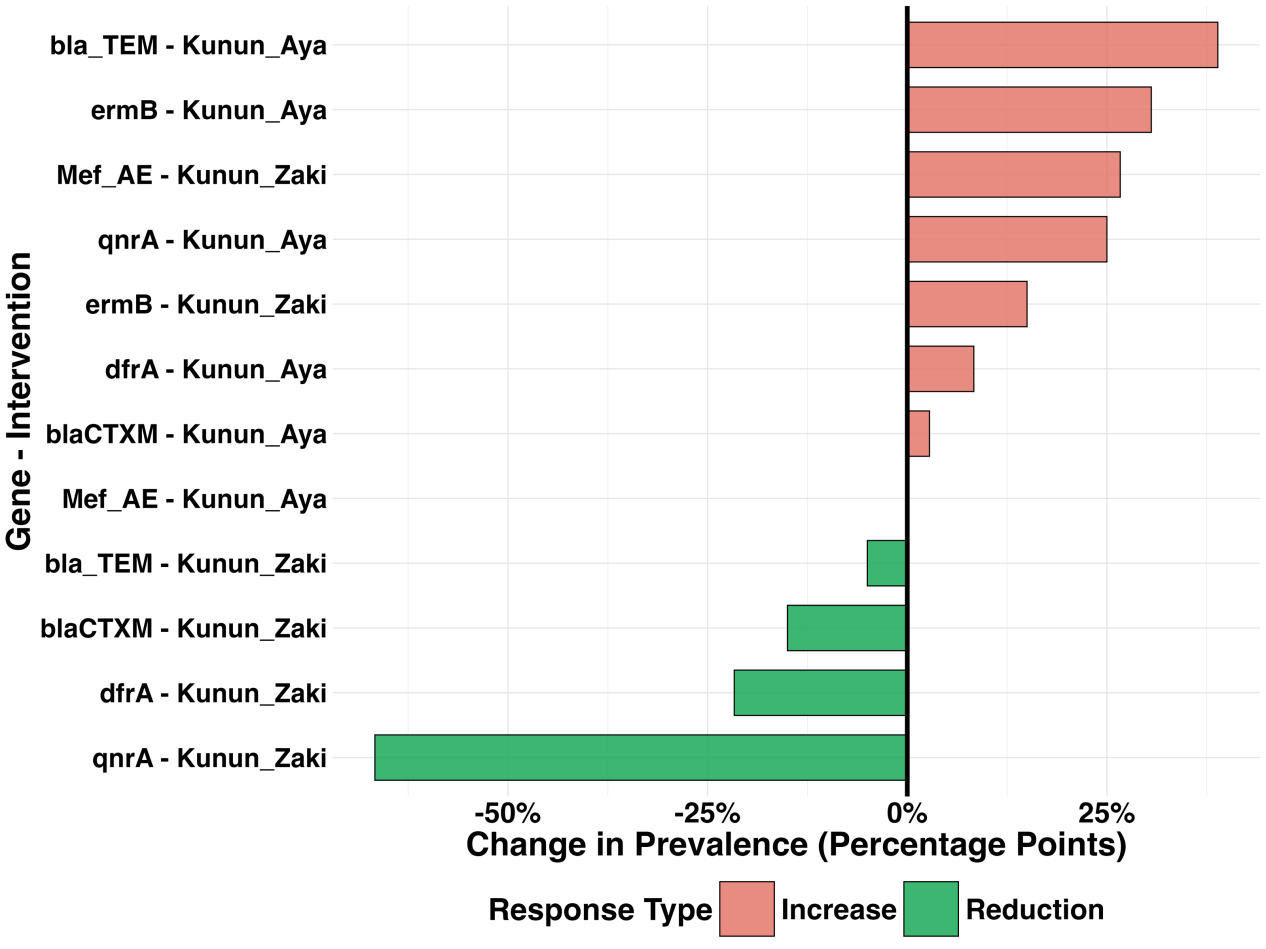
Individual gene responses. Bar chart showing change in prevalence (percentage points) from baseline to washout for each gene-intervention combination. Positive values (red bars) indicate increases in prevalence; negative values (green bars) indicate decreases.

The minimal directional change in the control group (mean: +0.8 percentage points) compared to the consistent reductions in the *Kunun zaki* group (mean: −11.1 percentage points) suggests that *Kunun zaki* consumption was associated with reductions in resistance gene prevalence rather than reflecting natural temporal variation.As such, *Kunun zaki* consumption was associated with reductions in resistance gene prevalence, with declines in four out of six tested genes and a mean reduction of −11.1 percentage points across all genes (Fig. 5). In contrast, *Kunun aya* was associated with increases in five out of six tested genes, with a mean rise of +17.6 percentage points. The control group showed minimal net change (+0.8 percentage points), suggesting that the directional shifts observed in the intervention groups reflect genuine beverage-associated effects rather than temporal variations.

### Gene co-occurrence patterns

**Figure 6:**
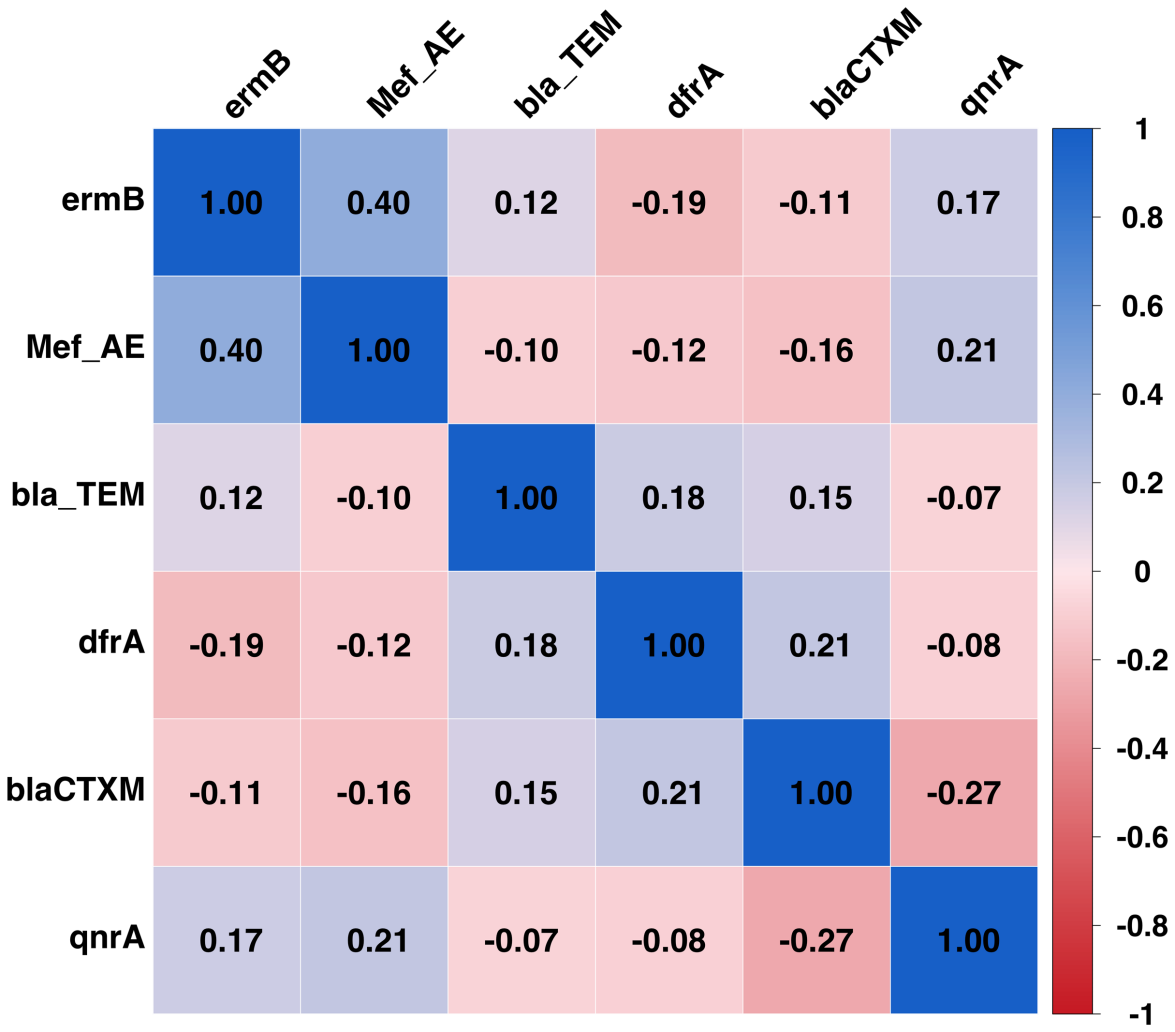
Gene occurrence corelation at baseline. Correlation matrix showing Pearson correlation coefficients (r) between the six ARGs at baseline (*n* = 36 participants). Color intensity indicates strength of correlation: blue for positive correlations, red for negative correlations. Numbers in cells represent correlation coefficients.

Gene co-occurrence analysis at baseline revealed predominantly weak correlations among resistance genes (Fig. 6). The strongest association was between *MefAE* and *ermB* (*r* = 0.40, *p* = 0.04). No gene pairs showed strong correlations (—r— ≥ 0.7)”.

### AMR gene distribution and association by sex

Analysis of ARG prevalence by sex at baseline revealed no statistically significant differences (Fisher’s exact test, all *p* > 0.05). Descriptive trends showed slightly higher prevalence in female participants for all six genes tested, with the largest differences observed for *MefAE* (47.4% *vs* 29.4%, *p* = 0.32) and *qnrA* (36.8% *vs* 23.5%, *p* = 0.48).

## Discussion

This exploratory pilot study investigated the distribution of gut antimicrobial resistance genes among participants who supplemented their diet with traditional Nigerian fermented beverages *Kunun aya* and *Kunun zaki*, in comparison to a control group. Among the 11 ARGs screened, *dfrA* was the most prevalent, followed by *blaTEM,* and then *mefA/E*. The high baseline prevalence of *dfrA* in our study is consistent with previous reports identifying this gene as a common resistance determinant in the gut microbiota. Notably, studies in community settings have also documented widespread carriage of *dfrA* among commensal *E. coli*. In Nigeria, Labar et al. (2012) found integron-borne *dfrA* in 43.1% of fecal *E. coli* isolates from healthy adults. Odetoyin et al. (2017) also reported class-1 integrons in 37.5% of mother–child fecal isolates, with *dfrA7* and *dfrA5* present in 18.3% and 14.4% of isolates, respectively. In Australia, Bailey et al. (2010) reported the presence of multiple *dfrA* variants (*dfrA1, dfrA5, dfrA7, dfrA12, dfrA17*) in commensal *E. coli* from healthy adults. Fadeyi et al. (2023) also reported the presence of *bla* TEM, *bla* SHV, *bla* CTX-M, *dfrA, tet*, *mef, ermB, ermA*, *blaZ* and *aac* genes in fecal samples of babies in Ekiti State, Nigeria. In China, Li et al. (2014) identified integrons in 26.7% of fecal *E. coli* isolates from healthy individuals. Compared to these community-level estimates, the prevalence in our study was markedly higher, particularly in the intervention groups at baseline, suggesting that *dfrA* is not only entrenched in the gut resistome but may also be shaped by dietary or ecological factors specific to the studied population. Furthermore, *dfrA i* s one of the genes that codes for trimethoprim resistance and trimethoprim is sold under the brand name Setprin in Nigeria. Septrin is one of the most abused and poorly regulated antibiotics in Nigeria (Ibrahim et al., 2020). In Nigeria, Makut et al. (2013) reported that 100% of *Escherichia coli* isolates from local samples exhibited multidrug resistance, with high resistance rates to chloramphenicol (75%), septrin (68.7%), and perfloxacin (68.7%). Similarly, the widespread presence of ermB and mefA/E, both conferring macrolide resistance, is consistent with global reports on rising macrolide resistance linked to their clinical use (Li et al., 2023; Schroeder & Stephens, 2016).

The current study revealed dynamic changes in AMR gene prevalence over time, particularly in response to fermented beverage interventions. Participants in the *Kunun zaki* group showed reductions in *qnrA* gene as well as reductions in the *dfrA* gene prevalence over time. These findings suggest that *Kunun zaki* may be associated with the modulation of the gut resistome, potentially through mechanisms such as competitive exclusion, acidification, and the action of bioactive metabolites produced by its lactic acid bacteria content (Evurani et al., 2019; Aderolake, Adeola & Amos, 2023; Oluwajoba, Akinyosoye & Oyetayo, 2013). Previous studies such as that by Ayeni et al. (2009), similarly reported the suppression of multi-drug-resistant Gram-negative pathogens by lactic acid bacteria, supporting the plausibility of our findings Changes in AMR prevalence may also reflect the bioactive properties of the constituent ingredients. Sorghum (*Sorghum bicolor*), the primary constituent of *Kunun zaki*, contains phenolic compounds and tannins with demonstrated antibacterial potential. A recent study demonstrated the *in-vitro* antimicrobial potential of *Sorghum bicolor* phenolic extracts on liver abscess causing bacterial pathogens (Salih et al., 2025). Ginger (*Zingiber officinale*) has been extensively studied for its antibacterial activity, with gingerol and shogaol compounds shown to suppress both Gram-positive and Gram-negative pathogens, and to weaken bacterial virulence factors (Shan et al., 2007; Ghasemzadeh, Jaafar & Rahmat, 2016). Similarly, cloves (*Syzygium aromaticum*), rich in eugenol, have demonstrated potent bactericidal activity against multiple strains, including multidrug-resistant *E. coli* and *Klebsiella pneumoniae* (Cortés-Rojas, De Souza & Oliveira, 2014; Nuñez & D’Aquino, 2012). Although dried potato (*Solanum tuberosum*) is less well characterized in this regard, phenolic acids present such as chlorogenic acid have been reported to exert moderate antibacterial activity against enteric bacteria (Friedman, 2006).

These findings are consistent with previous studies where fermented foods such as yogurt, kefir, and kimchi were associated with reductions in colonization by AMR-carrying bacteria and altered gene transfer dynamics (Leech et al., 2020; Qu et al., 2024; Li et al., 2024). The potential association between *Kunun zaki* consumption and reduced AMR gene prevalence highlights the relevance of traditional fermented foods as low-cost, accessible, and culturally accepted interventions in low- and middle-income countries (LMICs), where formal AMR control programs may be limited (Evurani et al., 2019; Yasir et al., 2023). Nwuche (2013) isolated bacteriocin-producing LAB from Ugba and Okpiye, two Nigerian condiments, and showed their activity against *Staphylococcus aureus, Listeria monocytogenes,* and *Escherichia coli*, suggesting a role in biopreservation and suppression of pathogens (Nwuche, 2013). Similarly, Alimi, Ogunbanwo & Alimi (2023) found LAB from fruits in Ibadan particularly *Lactiplantibacillus plantarum* and *L. brevis* produced organic acids, hydrogen peroxide, and diacetyl, with strong inhibition against multi-drug-resistant food-borne pathogens (Alimi, Ogunbanwo & Alimi, 2023). Ayeni et al. (2009) reported lactic acid bacteria isolated from Nigerian dairy products and cow intestines could inhibit multi-drug-resistant uropathogens. More recently, Nwachukwu et al. (2025) demonstrated that bacteriocins from *Lactobacillus plantarum* and *L. fermentum* isolated from fermented maize and cassava products significantly inhibited spoilage bacteria such as *Bacillus cereus, Pseudomonas aeruginosa*, and *Enterobacter aerogenes* (Nwachukwu et al., 2025). Interestingly *qnrA* showed a sharp decline with intervention in the Kunun Zaki group. The observed decline in qnrA prevalence may be indicative of shifts in the gut microbial community that reduce the persistence of qnrA-harboring organisms. This finding aligns with previous research demonstrating that fermented foods may modulate gut microbial ecology, potentially leading to the displacement of resistant strains and alterations in the gut resistome structure (Valero-Cases et al., 2020; Stiemsma et al., 2020; Choi et al., 2025).

In contrast, *Kunun aya* consumption was associated with increases in ARG prevalence likely reflecting differences in fermentation profiles, microbial diversity and lack of cereal constituents. Moreover, raw and commercially processed tiger nuts have been shown to sometimes contain diverse bacterial communities, including coliforms and *Enterobacteriaceae*, some of which exhibit antibiotic resistance such as cotrimoxazole resistance (Ayeh-Kumi et al., 2014). This may potentially contribute to the persistence and spread of antibiotic resistance determinants in consumers. Similarly, the nutrient profile of *Kunun aya* which is rich in simple sugars and lipids, may favor proliferation of Proteobacteria/Enterobacteriaceae in the gut. Studies have shown that high-sugar and high-fat diets increase the chance of horizontal transfer of exogenous ARGs among intestinal microbiota (Tan et al., 2022).

Sex had no significant impact on AMR gene prevalence (Fisher’s exact test, all *p* > 0.05). Descriptive trends showed slightly higher prevalence of all analyzed genes in female participants, with the largest differences observed for *MefAE* (47.4% *vs* 29.4%) and *qnrA* (36.8% *vs* 23.5%), though these differences did not reach statistical significance. The lack of statistical power due to modest sample size limits our ability to draw definitive conclusions about sex-based differences in ARG carriage. *Kunun zaki* consumption was associated with a tendency to reduce resistance burden, with four of six genes declining and an overall mean reduction of 11.7 percentage points was noteworthy. The fermentation process in *Kunun zaki* may foster beneficial microbial communities through competitive exclusion mechanisms, where lactic acid bacteria and other beneficial microorganisms outcompete resistance-harboring bacteria for essential nutrients and binding sites (Siedler et al., 2020). Conversely, *Kunun aya* is made up mainly of nuts and spices, this profile appears to create an environment conducive to resistance gene proliferation, which may explain its association with five of six genes increasing (mean rise: 18.1 percentage points) in this study. Recent metagenomic studies have demonstrated that fermented foods can serve as reservoirs of antimicrobial resistance genes, with some traditional fermented products containing diverse resistance determinants against multiple antibiotic classes (Ashaolu & Reale, 2020; Wolfe, 2023). The control group’s stability (overall mean change: +0.8 percentage points) reinforces that these directional shifts reflect genuine fermentation-associated effects rather than temporal variations. These findings underscore the duplibiotic nature of fermented foods as potential vehicles for both beneficial bacteria and antimicrobial resistance determinants (Walsh et al. 2016; Li et al., 2024).

In summary, the results suggest that fermented cereal foods like *Kunun zaki* may be associated with reductions in specific AMR genes in the human gut, particularly *dfrA* and *qnrA*, likely through microbiota modulation. These findings align with emerging literature demonstrating the potential of beneficial bacteria-rich diets to reduce AMR gene carriage *via* mechanisms such as microbial competition, gut acidification, and inhibition of horizontal gene transfer (Wastyk et al., 2021). Given their low cost, accessibility, and cultural acceptance, such dietary interventions offer a promising, sustainable approach for mitigating AMR burden in LMICs, especially in community settings with limited formal AMR stewardship programs

## Conclusion

This study provides preliminary evidence that traditional fermented beverages, particularly *Kunun zaki*, may be associated with reductions in the prevalence of specific antimicrobial resistance genes in the gut microbiota of humans. Importantly, the two beverages showed contrasting effects: while *Kunun zaki* consumption was associated with reductions in four of six genes, *Kunun aya* consumption was associated with increases in five of six genes, underscoring the fact that fermented foods have variable effects on the gut resistome depending on their composition and fermentation profile. These findings suggest the potential of culturally relevant dietary interventions as a complementary strategy to address the growing global AMR threat in low and middle income countries where such foods are widely consumed

## Study Limitations

First, the quasi-experimental design without proper randomization might be responsible for observed baseline imbalances. Second, the small sample size, though sufficient for microbiome studies, may have reduced statistical power for detecting moderate effect sizes and assessing gene-gene interactions. Third, this study employed conventional PCR to assess qualitative presence or absence of ARGs rather than quantitative PCR (qPCR) to measure gene copy number or relative abundance. While this approach was sufficient for our exploratory aims it does not capture quantitative changes in gene load. Future studies should consider qPCR or even shotgun to capture the full picture of gene dynamics post intervention. Fourth while we discussed the potential for fermented foods to serve as both suppressors and reservoirs of ARGs, we did not directly characterize the microbial composition or ARG content of the beverages consumed in this study.

## Supplemental Information

10.7717/peerj.21495/supp-1 Supplemental Information 1 Raw data. Each Column represents the results for each gene, with attached suffixes indicating the timepoint for each gene (dfrA_01, dfrA_02, dfra_03 shows timepoints 1, 2 and 3 respectively). Gene presence or absence is indicated by 0 (absent) and 1 (present). NA indicates absence of data for that participant for that time point. The R codes used for the analysis are available in the Zenodo repository.

10.7717/peerj.21495/supp-2 Supplemental Information 2 Codebook.

10.7717/peerj.21495/supp-3 Supplemental Information 3 Representative gel image showing blaTEM with controls Image shows blaTEM gene electrophoresis result. Presence and absence of genes were recorded for individuals corresponding to the reflected code. This is detailed in the raw data sheet.

10.7717/peerj.21495/supp-4 Supplemental Information 4 Consort checklist.

10.7717/peerj.21495/supp-5 Supplemental Information 5 Trial protocol.

## Additional Information and Declarations

### Data Availability

The following information was supplied regarding data availability: The data is available in the Supplemental Files. The data and code are available at Zenodo: Ayeni, F., Audu Jimoh, H., Byonanebye, M., Allen, N., & ALABI, O. S. (2026). Potential of Human consumption of fermented millet (Kunun zaki) to reduce the prevalence of selected antimicrobial resistance genes in human fecal samples. Zenodo. https://doi.org/10.5281/zenodo.18639580.
