# Supplementary material for: Potential for human consumption of fermented millet (Kunun zaki) to reduce the prevalence of selected antimicrobial resistance genes in human fecal samples
Source: PeerJ. 2026 Jul 14;14:e21495. doi: 10.7717/peerj.21495 (PMC13378467; doi:10.7717/peerj.21495)
Supplement: Supplemental Information 2 [file peerj-14-21495-s002.docx]

**DATA DICTIONARY**

**Participant codes:**

Participants were assigned codes in the format XYY, that represented the following:
X: Participant Group (A, or B, or C)

YY: Participant ID (A01 = Participant 01 in Group A)

**Sample timepoints**

The time point of collection were indicated by suffixes attached to gene names.

01 indicated timepoint 1 (dfrA_01 indicated dfrA prevalence at timepoint 1....)

02 indicated timepoint 2

03 indicated timepoint 3

**Gene Presence/Absence reporting**Gene presence was represented by the number 1

Gene absence was represented by the number 0

NA indicated participant had no record for that timepoint
