# Supplementary figures and images for: Potential for human consumption of fermented millet (Kunun zaki) to reduce the prevalence of selected antimicrobial resistance genes in human fecal samples
Source: PeerJ. 2026 Jul 14;14:e21495. doi: 10.7717/peerj.21495 (PMC13378467; doi:10.7717/peerj.21495)

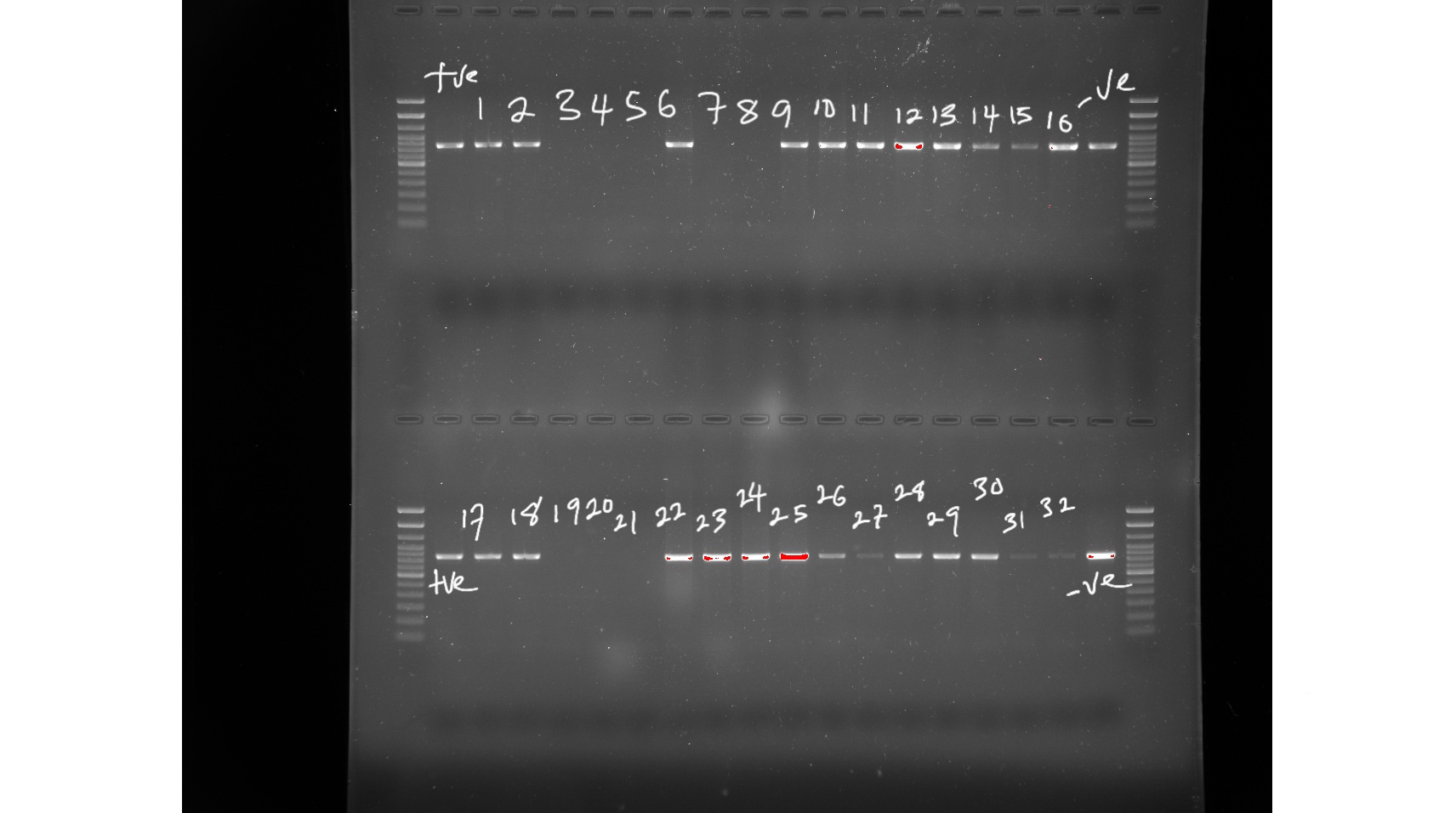

Supplement: Supplemental Information 3 — Image shows blaTEM gene electrophoresis result. Presence and absence of genes were recorded for individuals corresponding to the reflected code. This is detailed in the raw data sheet. [file peerj-14-21495-s003.jpg]
