# Supplementary material for: Potential for human consumption of fermented millet (Kunun zaki) to reduce the prevalence of selected antimicrobial resistance genes in human fecal samples
Source: PeerJ. 2026 Jul 14;14:e21495. doi: 10.7717/peerj.21495 (PMC13378467; doi:10.7717/peerj.21495)
Supplement: Supplemental Information 5 [file peerj-14-21495-s005.docx]

**Human consumption of fermented millet (*Kunun zaki*) reduces the prevalence of selected antimicrobial resistance genes in human fecal samples**

Haruna Jimoh Audu^1,4,^, Marion Byonanebye^2,4^, Nyah Allen^,3 ,^ Olumuyiwa Samuel Alabi.^1^, Funmilola A. Ayeni^2,5^

1. ^Pharmaceutical Microbiology and Biotechnology, Faculty of Pharmacy, University of Ibadan, Ibadan, Oyo State, Nigeria^
2. ^Environmental and Occupational Health, School of Public Health, Indiana University Bloomington, Indiana, USA^
3. ^Biology, Indiana University Bloomington, Indiana, USA^
4. ^These authors had equal contributions.^
5. ^Corresponding author (fayeni@iu.edu)^

# SUMMARY OF RESEARCH

**TITLE: Human consumption of fermented millet (*Kunun zaki*) reduces the prevalence of selected antimicrobial resistance genes in human fecal samples**

**BACKGROUND:** The gut microbiota is home to more than 500 philotypes or approximately 1 million bacterial genes. It provides a secondary barrier against pathogenic colonisation. It is also host to neurons hormones and secondary messengers (largest neuroendocrine organ in the body) and therefore regulates many physiological functions. In the same way a dysbiosis of the gut microbiota could result in proliferation of pathogenic strains of bacteria and impair the physiologic functions of the body, it could also be deliberately modulated to achieve beneficial effects through competitive exclusion. This has been demonstrated in many researches just as whole cereal grain have been shown to have potential health effects.

The gut microbiota serves as a reservoir for antimicrobial resistance genes (ARGs), which can be transferred between commensal and pathogenic bacteria. Understanding how dietary interventions, particularly traditional fermented beverages, modulate the gut resistome is crucial for developing strategies to reduce antimicrobial resistance burden in communities.

**Study Population and Sample Collection**

A total of 36 healthy human volunteers (males and females), aged between 3 and 65 years and residing in Abuja, North Central Nigeria, will be recruited for this study. Eligible participants will enter a two-week monitoring phase to ensure compliance with exclusion criteria, verified through a daily online 24-hour food and drug recall questionnaire. Participants will then be assigned to Kunun aya, Kunun zaki, or control group (no intervention) by chance as they come, with 12 participants per group. Participants will supplement their habitual diet with 500 mL daily of the assigned beverage for two weeks, followed by a two-week washout phase.

Fecal samples will be self-collected by participants across three phases: prior to beverage consumption (baseline), after two weeks of beverage consumption, and at two weeks post-intervention (washout phase). Samples will be prepared according to the method described by Nsubuga et al. (2004). DNA will be extracted from fecal samples using the DNA Stool Mini Kit (Qiagen, USA) according to the manufacturer's instructions and stored at −20°C.

**Expected Outcome:** At the end of this research it is expected that there will be significant changes in the gut resistome composition of participants following consumption of the local drinks. Specifically, we expect to observe changes in the abundance and diversity of antimicrobial resistance genes, with potential decreases in clinically relevant ARG classes through competitive exclusion mechanisms and modulation of the gut microbial community structure.

**Protocol and Statistical Analysis Plan Access**

The full research protocol and statistical analysis plan will be made publicly available through the research registry and the institutional repository of the University of Ibadan upon completion of ethics approval. Additional materials may be obtained by contacting the principal investigator at the Department of Environmental and Occupational Health, School of Public Health, Indian University

# Data Sharing Statement

The datasets generated and analyzed during the current study are available from the corresponding author upon reasonable request. De-identified participant data, the data dictionary, and statistical code will be shared following approval of a proposal and with a signed data access agreement.

# Funding and Conflicts of Interests

## Funding Sources

No funding sources are reported for this research

## Conflicts of Interest

The authors declare no financial or other conflicts of interest related to this research.

# **INTRODUCTION**

**Background of the Study**

In recent years, there has been increased focus by researchers on the composition and relative abundance of the gut Microbiota. With improving technologies that allow whole genome sequencing, metagenomics analysis of the gut microbiota has revealed better insights and holds promising future for the field of Microbiology and Medicine. The gut Microbiota is a rich flora of bacteria that naturally inhabit the gut playing significant role in metabolism among many other functions (Cani 2018). Because of their inter-relationship with different human cell, changes in their composition and distribution have been linked to potential diseases and even disease symptoms (Cani 2018).

The gut microbiota is home to more more than 500 philotypes or approximately 1 million bacterial genes (Jiang et al 2013). The gut microbiota not only provides a secondary barrier against pathogenic colonisation but is also a host to neurons hormones and secondary messengers (largest neuroendocrine organ in the body) and therefore regulates many physiological functions (Neuman et al 2015). It also produces Metabolites like SFA, provides energy to epithelial cells of intestine and prevents the expression of virulence factors (Rinttila and Apajalahti 2013)

In the same way a dysbiosis of the gut microbiota could result in proliferation of pathogenic strains of bacteria and impair the physiologic functions of the body, it could also be deliberately modulated to achieve beneficial effects through competitive exclusion. This has been achieved in the case of newly hatched chicks prevented from Salmonella enteritidis infection through administration of suspension of intestinal contents of healthy chickens to the chicks in doses (Kogut 2019). Gargari et al (2016) investigated gut microbiota modulation through the consumption of a Bifidobacterium bifidum strain for 4 weeks and found that the dominant intestinal bacterial taxa and fecal butyrate in healthy adults were modulated. While attempts to modulate the gut microbiota usually focuses on direct repopulation of gut microbiota with beneficial organisms, there have been also indications that certain food substances provide nutrients that help in developing and maintaining a healthy gut microbiota population. For instance, whole cereal grain are said to have potential health effects (Gong et al 2018). In a 6 week randomized research, adults who consumed whole grains had their gut microbiota as well as immune and inflammatory markers positively impacted (Vanegas et al, 2017). In a 4 weeks randomized cross over research, healthy humans who consumed whole grains of barley and brown rice, lead to an increase in microbial diversity (Martinez et al, 2013).

The gut microbiota also serves as a major reservoir for antimicrobial resistance genes (ARGs). These genes can be transferred horizontally between commensal and pathogenic bacteria, contributing to the spread of antimicrobial resistance. The gut resistome encompasses all resistance genes present in the gut microbiota, including those conferring resistance to clinically important antibiotics such as beta-lactams, macrolides, tetracyclines, and aminoglycosides. Dietary interventions that modulate gut microbial composition may consequently affect the abundance and diversity of resistance genes in the gut ecosystem.

### Rationale for the Study

Kunu contains beneficial bacteria as well as blended cereal (fibre) which makes it suitable to serve both as a probiotic through gut microbiota modulation, and prebiotic by stimulating the growth of other bacteria. The fermentation process in Kunun production involves lactic acid bacteria which may competitively exclude potential pathogenic bacteria and their associated resistance genes. Understanding how traditional fermented beverages affect the gut resistome is important for public health, particularly in regions where antimicrobial resistance is a growing concern and where these beverages are regularly consumed.

### **Objective of the study**

The general objective of this research is to study possible gut resistome changes due to consumption of local drinks for a period of two weeks, comparing gut resistome profile before, during and after the period through:

1. Analysis and comparison of gut resistome changes in participants after consumption of Kunu zaki and Kunu aya for a period of two weeks.

2. Study specific antimicrobial resistance gene signatures that may appear or disappear after Kunu consumption.

3. Evaluate changes in the abundance and diversity of clinically relevant resistance genes following consumption of Kunu Zaki and Kunu Aya for two weeks.

## Primary and Secondary Outcomes

### Primary Outcome

The primary outcome is the change in gut resistome composition, measured as the difference in total antimicrobial resistance gene (ARG) abundance and diversity between baseline and post-intervention timepoints (2 weeks). This will be quantified using ARG detection by PCR, with results analyzed at the conclusion of the 2-week intervention period. The primary endpoint compares ARG profiles between the intervention groups (Kunun Zaki and Kunun Aya) and the control group using permutational ANOVA (PERMANOVA) with alpha set at 0.05.

### Secondary Outcomes

1. Prevalence changes of specific ARG classes (beta-lactam, macrolide, tetracycline, aminoglycoside resistance genes) measured at baseline, 2-week post-intervention, and 2-week washout periods.

2. Persistence of resistome changes during the washout phase (measured at 2 weeks post-intervention cessation) compared to baseline and intervention periods.

3. Identification of specific ARG signatures that appear or disappear following consumption of each fermented beverage.

4. Safety outcomes including participant-reported gastrointestinal symptoms and adverse events throughout the study duration.

# METHODS

**Patient and Public Involvement**

Patients and members of the public were not involved in the design, conduct, or reporting plans for this research. However, participants will be informed of the study results upon completion through a summary report and will have the opportunity to receive their individual resistome profile results if they express interest.

**Research Design**

This is a human intervention study with three arms (1:1:1 allocation ratio) designed to assess superiority of two fermented beverage interventions (Kunun Zaki and Kunun Aya) compared to a control group (no intervention) in modulating the gut resistome. The research follows a quasi-experimental design with participants assigned to different groups by chance to receive either *Kunun Zaki, Kunun Aya*, or no intervention for a 2-week period, followed by a 2-week washout phase. Sample collection occurs at baseline (pre-intervention), at the end of the 2-week intervention period, and at the end of the 2-week washout period.

**Research Setting**

Study site: All samples will be collected from residents of Abuja, North Central Nigeria. All laboratory analysis will be carried out in Prof. Funmilola Ayeni’s Laboratory at the Department of Environmental and Occupational Health, School of Public Health, Indiana University.

**Study population**

A total of 36 persons will be selected for this study (12 per group).

### Eligibility Criteria

### Inclusion criteria

Healthy individuals aged between 3 and 65 years, good general health, and a signed consent form (or assent with parental consent for minors).

### Exclusion criteria

Engaging in any of these during 2 weeks prior to the first sample collection; antibiotic use, intentional intake of probiotic or prebiotic products, presence of gastrointestinal disorders such as diarrhea, inflammatory bowel disease, or irritable bowel syndrome, pregnancy or breastfeeding, alcoholism and drug addiction. For the entire 6 weeks this research will last for, participants will be required to report their daily food and drug intake using an individualised web app developed for this purpose.

Interventions and Comparator

**Intervention 1 - Kunun Zaki:** Participants will consume 500 mL of Kunun Zaki daily for 2 weeks. Kunun Zaki is a traditional Nigerian fermented beverage made from millet or sorghum, supplemented with ginger and cloves as spices.

**Intervention 2 - Kunun Aya:** Participants will consume 500 mL of Kunun Aya daily for 2 weeks. Kunun Aya is a traditional Nigerian fermented beverage made from tiger nuts, prepared through similar fermentation processes as Kunun Zaki.

**Control:** Participants in the control group will maintain their habitual diet without any intervention beverage for the 2-week period.

All participants will be instructed to maintain their usual dietary habits aside from the intervention beverage. Interventions will be delivered by study personnel who will ensure participants receive fresh beverages daily. Adherence will be monitored through daily self-reported logs via the web-based food diary.

**Sample Size**

A sample size of 36 participants (12 per group) was determined based on feasibility considerations and previous microbiome intervention studies.

### Laboratoy Methods

### DNA Extraction

DNA will be extracted from fecal samples using the DNA Stool Mini Kit (Qiagen, USA) following the manufacturer's instructions. Extracted DNA will be quantified using a NanoDrop spectrophotometer and stored at −20°C until analysis.

### Antimicrobial Resistance Gene Detection

Target antimicrobial resistance genes will be detected using conventional PCR with specific primer sets for genes conferring resistance to beta-lactams (blaTEM, blaSHV, blaOXA), macrolides (ermB, ermC, mef(A/E)), tetracyclines (tetA, tetB, tetM), and aminoglycosides (aac(3)-II, aac(6')-Ib, aph(3')-IIIa). Multiplex PCR will be employed where feasible to detect multiple genes simultaneously. Both multiplex and singleplex PCR reactions will be conducted in 20 μL volumes containing Master mix, gene-specific primers, DNA template, and molecular-grade water. Cycling conditions will be optimized for each gene target. Positive controls and negative controls (molecular water) will be included in all PCR runs to validate results.

**Data Collection procedure**

Sample collection would be done according to the method described by Nsubuga et al., (2004). Participants will be given sample bottles to collect their faecal sample after proper tutelage on the collection procedure. Approximately 5g of participant's feces would be aseptically collected into a tube containing 30 ml of 97 % ethanol and allowed to stand for 24-36 h. The bolus formed afterwards will then be transferred to 50ml tubes containing silica gel beads topped with a kim wipe and then stored at room temperature.

### Frequency of interviews

Interviews will be in the form of questionnaires used to anonymously collect data on their suitability as participants. There will be no other interviews besides the first contact and the 24hr food and drug recall.

### Statement on invasive sampling

No invasive samples will be collected from the participants.

### Statistical methods for primary and secondary outcomes

ARG prevalence will be calculated for each group at baseline, post-intervention, and washout phases. Statistical comparisons between groups and across time points will be performed to determine the effect of beverage consumption on antimicrobial resistance gene patterns in the gut microbiota.

Statistical comparisons of ARG prevalence between groups will be conducted using appropriate parametric or non-parametric tests depending on data distribution. Significant differences in resistome composition will be determined using permutational ANOVA (PERMANOVA) at 95% Confidence Interval and 0.05α. Comparison of means will be done using an unpaired t-test and Tukey's multiple-comparisons test where appropriate.

ARGs will be categorized by resistance mechanism and antibiotic class to identify which categories are most affected by the intervention. Changes in clinically relevant resistance genes will be given particular attention in the analysis and interpretation of results.

### Participants included in analysis

All participants with complete data will be used for this study.

### Handling of missing data

Missing data will be documented and reported. For the primary outcome, participants with missing post-intervention samples will be excluded from the per-protocol analysis but included in intention-to-treat analysis using last observation carried forward where appropriate.

**Ethical Consideration**

This research intends to simulate as close to natural as possible the habitual consumption of the local drinks by the participants, however there remains the risk of possible contamination of prepared drinks during handling by local sellers posing a limited threat to the health of participants (limited because they are used to consuming it ab initio from such sources). Participants will be directed to report any symptom of food poisoning for treatment and discontinuation of research for that participant, as well as other necessary precautions. There is also the concern about handling fecal matter for which participants will be trained on aseptic handling of fecal material, and be provided appropriate materials for same such as sample containers with an attached scoop for easier sample collection, and gloves.

**Confidentiality of data -** The sample bottles would be coded as simple as possible and will not be traceable to any of the volunteers. Names of subjects or identifiers will not be used in any publication or reports from this research. Any personal information collected during the research will be considered and treated as confidential.

**Translation of protocol to the local language -** the protocol will be translated to the local language of the subjects (Hausa) for few persons who may not understand English.

**Beneficience to participants -** After the research, participants will be able to know the positive impact of their diet type on their gut microbiota and by implication food digestion and absorption. Participants will also gain knowledge about antimicrobial resistance genes present in their gut microbiota.

**Non maleficence to participants -** the research will be conformed to professional standard thereby providing no risk to participants.

**Voluntariness --** participation is entirely voluntary

# REFERENCES

Ayeni, F. A., Biagi, E., Rampelli, S., Fiori, J., Soverini, M., Audu, H. J., ... & Turroni, S. (2018). Infant and adult gut microbiome and metabolome in rural Bassa and urban settlers from Nigeria. Cell reports, 23(10), 3056-3067.

Gargari, G., Taverniti, V., Balzaretti, S., Ferrario, C., Gardana, C., Simonetti, P., & Guglielmetti, S. (2016). Consumption of a Bifidobacterium bifidum strain for 4 weeks modulates dominant intestinal bacterial taxa and fecal butyrate in healthy adults. Applied and environmental microbiology, 82(19), 5850-5859.

Gong, L., Cao, W., Chi, H., Wang, J., Zhang, H., Liu, J., & Sun, B. (2018). Whole cereal grains and potential health effects: Involvement of the gut microbiota. Food research international, 103, 84-102.

Jiang, W., Wang, X., Zeng, B., Liu, L., Tardivel, A., Wei, H., ... & Zhou, R. (2013). Recognition of gut microbiota by NOD2 is essential for the homeostasis of intestinal intraepithelial lymphocytes. Journal of Experimental Medicine, 210(11), 2465-2476.

Kogut, M. H. (2019). The effect of microbiome modulation on the intestinal health of poultry. Animal feed science and technology, 250, 32-40.

Martínez, I., Lattimer, J. M., Hubach, K. L., Case, J. A., Yang, J., Weber, C. G., ... & Walter, J. (2013). Gut microbiome composition is linked to whole grain-induced immunological improvements. The ISME journal, 7(2), 269-280.

Neuman H., Debelius J. W., Knight R., Koren O. (2015). Microbial endocrinology: The interplay between the microbiota and the endocrine system. FEMS Microbiology Reviews, 39, 509--521. doi:10.1093/femsre/fuu010

Nsubuga AM., Robbins MM., Roeder A., Morin P., Boesch C. and Vigilant L. 2004. Factors affecting the amount of genomic DNA extracted from ape feces and the identification of an improved sample storage method. Molecular Ecology. 13: 2089-2094.

Rinttilä, T., & Apajalahti, J. (2013). Intestinal microbiota and metabolites--Implications for broiler chicken health and performance. Journal of Applied Poultry Research, 22(3), 647-658.

Vanegas, S. M., Meydani, M., Barnett, J. B., Goldin, B., Kane, A., Rasmussen, H., ... & Meydani, S. N. (2017). Substituting whole grains for refined grains in a 6-wk randomized research has a modest effect on gut microbiota and immune and inflammatory markers of healthy adults. The American journal of clinical nutrition, 105(3), 635-650.
